# Factors Associated with Outcomes of Non-Invasive Ventilation in Acute Hypercapnic Respiratory Failure with Acidosis: A Study from a Tertiary Care Hospital in Pakistan

**DOI:** 10.3390/jcm15051701

**Published:** 2026-02-24

**Authors:** Asifa Karamat, Shazia Akram, Huma Batool, Atiqa Ambreen, Tehmina Mustafa

**Affiliations:** 1 Department of Pulmonology, Gulab Devi Teaching Hospital, Lahore 54000, Pakistan; asifa.karamat@gmail.com; 2 Department of Pulmonology, Mohiuudin Islamic Medical College, Mirpur Azad Kashmir 10200, Pakistan; shazia_akram71@yahoo.com; 3 Department of Pulmonology, Jinnah Hospital, Lahore 54550, Pakistan; humabatool3@gmail.com; 4 Department of Microbiology, Gulab Devi Teaching Hospital, Lahore 54000, Pakistan; atiqaambren@gmail.com; 5 Center for International Health, Department of Global Public Health and Primary Care, University of Bergen, 5020 Bergen, Norway; 6 Department of Thoracic Medicine, Haukeland University Hospital, 5021 Bergen, Norway

**Keywords:** non-invasive ventilation, type 2 respiratory failure, respiratory acidosis, NIV failure, NIV outcome, mortality, hypercapnic respiratory failure

## Abstract

**Background**: This study aimed to identify factors influencing non-invasive ventilation (NIV) outcomes in patients with hypercapnic respiratory failure due to various respiratory conditions in a resource-limited intensive care unit (ICU) setting. These predictors can guide us in the prompt decision of ventilation, resulting in better outcomes. **Methods**: Patients requiring NIV for hypercapnic respiratory failure of any cause were included. Arterial blood gases were measured at 1 and 24 h, and an improvement in pH ≥ 7.35 was taken as a cut-off for early and late physiological responses, respectively. Binary regression analysis was used to identify predictors of physiological response, need for mechanical ventilation, and mortality. **Results**: Among 226 patients (139 males), the underlying causes were obstructive (71%), restrictive (25%), and infective disorders (4%). Older age, higher one-hour PCO_2_, FiO_2,_ and respiratory rate were associated with increased mortality. Late physiological response correlated with higher IPAP and WBC counts, while higher WBC counts also predicted need for mechanical ventilation on binary logistic regression. **Conclusions**: Higher one-hour PCO_2_, older age, higher FiO_2_, respiratory rate, WBC count, and IPAP predicted an unfavorable outcome of NIV in acute hypercapnoic respiratory failure. Locally generated data can support timely escalation to mechanical ventilation and inform patient selection for initial NIV therapy in resource-limited settings.

## 1. Introduction

Non-invasive ventilation (NIV) is a mode of assisted ventilation that delivers positive pressure throughout the respiratory cycle with additional phasic increases in airway pressure, without an endotracheal tube. Depending on the delivery system, these additional phasic increases in airway pressure can be synchronized or non-synchronized. NIV was successfully used for the management of acute respiratory failure (ARF) in chronic obstructive lung disease (COPD) patients in 1989, and ever since, it has been increasingly used in managing ARF in several other diseases [1].

ARF is a commonly encountered presentation to the emergency departments and intensive care units (ICUs), and one of the major causes of mortality among all age groups and genders [2]. Use of NIV has considerably reduced the need for mechanical ventilation among ARF patients, thereby reducing the complications related to invasive ventilation [3,4]. NIV has been shown to have better outcomes (reduced inpatient mortality and length of stay) when compared to invasive mechanical ventilation in randomized clinical trials, observational cohorts, or meta-analyses [5,6,7,8]. It is now the first line of therapy in emergency wards and ICUs for ARF encountered in acute exacerbation of COPD [3]. It is also extensively used in other indications, like pulmonary edema, interstitial lung disease, and acute respiratory distress syndrome [9]. There is also reasonable evidence of its use in COVID-19 respiratory failure, with improved patient outcomes [10]. A survey carried out in the United States showed a 400% increase in the use of NIV in one decade for acute exacerbation of COPD and 42% reduction in the use of invasive mechanical ventilation [11]. There is a paucity of data on the usage of NIV in low-income countries, including Pakistan. Secondly, most of the studies from low-income countries provide information regarding NIV in COPD and pneumonia patients, and data on other disorders causing respiratory failure are lacking. Local guidelines in Pakistan recommend it as a first-line therapy in ARF due to COPD [12,13]. The first study on NIV from a tertiary care hospital in Karachi, Pakistan, was published in 2010, evaluating its safe use and factors associated with NIV failure in both ICU and ward settings [14]. Later studies have shown the safe and effective use of NIV for the management of ARF as a first-line therapy, and a reduction in the need for mechanical ventilation, making it a cost-effective strategy [15]. However, these studies mainly included patients with COPD and pneumonia, and there is a scarcity of information on the efficacy of NIV in managing respiratory failure in different respiratory ailments in low-income ICU settings.

NIV failure is associated with higher in-hospital mortality and prolonged hospital and critical care stay [16]. It is essential to timely identify the risk factors leading to NIV failure for an appropriate intervention with endotracheal intubation and mechanical ventilation [17,18,19], or an assessment of prognosis and transition to palliative care. A team-based approach to NIV therapy is required for continuous assessment and early recognition of failure. The identification of key factors associated with NIV failure makes multidisciplinary teams work more smoothly and effectively, enabling timely interventions and improved patient outcomes [20]. Earlier studies have identified factors such as low initial pH, higher PCO_2_, high oxygen demand, weak cough reflex, depressed consciousness levels, and hospital-acquired pneumonia associated with NIV failure [14,21]. Similar factors also contributed to mortality. However, these studies are relatively small and included only COPD-induced ARF; many other underlying etiologies have not been studied adequately.

Patients with mild to moderate acidosis respond best to NIV treatment before the development of severe respiratory acidosis [22,23,24]. Established criteria for patients’ selection for NIV treatment include persistent acidosis or tachypnea despite optimal bronchodilators and controlled oxygen therapy [25]. Despite these standardized criteria and increasing experience with NIV therapy outside of the critical care setting, the rate of failure of NIV therapy is still reported to be as high as 20–30% [25,26,27]. In low-income countries like Pakistan, this failure risk is even higher due to a lack of trained staff and equipment [28]. Identification of the factors associated with favorable or unfavorable response to NIV in the ICUs is essential to refine criteria for timely initiation and termination/withdrawal of NIV, to ensure NIV is given to the most appropriate patients, thus improving the outcomes [19]. As the healthcare system in resource-limited countries such as, Pakistan lacks sophisticated critical care capabilities, investing in research on effective application strategies for NIV not only promises better patient management but also aligns with global standards.

The purpose of this study was to identify the factors predicting both early (1 h) and late (24 h) physiological responses to NIV, mortality, and need for mechanical ventilation in patients with acute hypercapnic respiratory failure, and highlight their relevance to clinical practice.

## 2. Materials and Methods

A retrospective analysis of the patients admitted with type II respiratory failure at the respiratory ICU of Gulab Devi Hospital (GDH), from July 2018 to July 2020, was performed. The data was accessed on first August 2020. GDH is a private not-for-profit tertiary care hospital located in Lahore, the capital of the largest populated province in Pakistan. The thirty-two-bed respiratory ICU provides services to patients with different respiratory diseases. The diagnosis of type II respiratory failure was based on the arterial blood gases (ABGs) done at the admission of the patients. X-rays and laboratory findings noted at the time of presentation were included in the study.

### 2.1. Inclusion Criteria

Patients with pH ≤ 7.35 and/or PaCO_2_ > 45 mmHg with at least one of these two symptoms,

(1) massive use of accessory muscles, (2) respiratory rate > 20 breaths/min.

### 2.2. Exclusion Criteria

Patients who refused the NIV trial or had any contradiction to NIV use were not included in the study.

### 2.3. NIV Protocol and Settings

We used pressure support ventilation or pressure-controlled ventilation using a full-face mask. BiPAP (Philips C series INTL 30 machines) were used. NIV was delivered to patients in bed at an angle of 30–45 degrees with a face mask. At the outset, the patients were started on an inspiratory positive airway pressure (IPAP) of 8 cm H_2_O and expiratory positive airway pressure (EPAP) of 4 cm H_2_O. The pressures were gradually adjusted as tolerated based on continuous pulse oximetry, arterial blood gases, and improvement in patients’ respiratory rate and distress. The duration of NIV and the time to stop NIV were determined based on arterial blood gas values. Oxygen was delivered by conventional devices (mask, non-rebreather, or nasal prongs) and flow rates were documented. The fraction of inspired oxygen (FiO_2_) was calculated by standard charts. Escalation and de-escalation were decided by bedside clinical response.

All patients were assessed at one hour and then after 24 h, and arterial blood gases were repeated.

### 2.4. Definitions of Patient Groups and Variables

Early physiological favorable response was defined as the normalization of arterial blood pH (≥7.35) or an increase in pH ≥0.05 and a decrease in PCO_2_ ≥ 10 mmHg at one hour. Late favorable physiological response was defined as the normalization of pH (≥7.35) at 24 h. These definitions are based on previous literature and local guidelines [29,30].

### 2.5. Disease Categories

Patients who were already diagnosed with COPD, asthma, and bronchiectasis were considered in the obstructive disease category. The current spirometry results of all patients were not available. Patients with CT evidence of interstitial lung disease, post-tuberculosis fibrosis, and kyphoscoliosis were included in the restrictive lung disease group. Patients with pneumonia not have any background obstructive or restrictive disease and radiological evidence of consolidation were considered in the infectious etiology group. Infective exacerbation of COPD or ILD (mixed phenotypes) was categorized in the obstructive and restrictive group and was not included in the infective group.

### 2.6. Statistical Analysis

SPSS version 26 was used for statistical analysis. Comparisons between two groups for continuous variables were performed using the Mann–Whitney U test. Comparisons among more than two groups were conducted using the Kruskal–Wallis test. Categorical variables were analyzed using the chi-square test. Outcomes assessed included early response, late physiological response, mortality, and survival.

Binary logistic regression analysis was performed to identify the factors associated with late physiological responses, the need for mechanical ventilation, and mortality.

For the late physiological response, the following variables were included: age, initial pH, initial PCO_2_, initial PO_2_, initial HCO_3_, one-hour PCO_2_, IPAP, FiO_2_, PO_2_/FiO_2_ ratio, respiratory rate, systolic blood pressure, white blood cell count, disease categories, and early favorable responses were included in the regression model. The regression models for the need for mechanical ventilation and mortality included 24 h pH, PCO_2_ values, as well as late physiological response.

Missing data were minimal (<2% for all variables). Analyses were therefore performed using complete-case analysis without imputation.

Multicollinearity among covariates was assessed using variance inflation factors (VIF). Most variables had VIF values < 3; only PO_2_ and PO_2_/FiO_2_ had VIF~5 but were retained in the regression analysis due to clinical significance in respiratory failure and disease severity.

## 3. Results

A total of 226 patients were included in the study; out of these, 139 (62%) were males. The majority, 71% (160/226), had obstructive lung disease, 25% (56/226) had restrictive lung disease, and 4% (10/226) had an infective etiology. Table 1 shows the demographic and clinical characteristics of patients stratified according to the disease category. Patients with obstructive and infective disorders had a higher median age (60 years) than patients with restrictive disorders (45 years) (*p* = 0.001). A significant male predominance was seen in obstructive disorders, whereas a female predominance was seen in restrictive disorders (*p* < 0.001). The history of smoking was more common (64%) in patients with obstructive as compared to those with restrictive (25%), and infective (20%) disorders (*p* < 0.001). Patients with infective etiology had a higher median pulse rate (104/min, but it was not statistically significant; however, respiratory rate (28/min) was significantly higher in patients with infective disorders (*p* = 0.004). Patients with obstructive disorders had lower FiO_2_ (*p* = 0.010), but higher initial PCO_2_ (*p* = 0.050) than the other two groups. Bicarbonate levels were lowest in the infective group as compared to the restrictive and obstructive disorders (*p* = 0.020). The most common radiological pattern was hyperinflation among patients with obstructive disorders, followed by consolidation and fibrosis, while hyperinflation was not seen in the other patient categories. Pneumothorax was observed in three patients on presentation. The intercostal drain was placed before the application of NIV. Overall mortality was seen in 37/226 (16%) patients. The patients with infective conditions showed significantly (*p* = 0.008) higher mortality (50%) as compared to the patients with obstructive (16%) and restrictive diseases (11%).

Echocardiography data were available for a total of 109 patients, showing pulmonary hypertension and cor pulmonale in 79 patients, and these conditions were more prevalent in obstructive and restrictive disorders.

**Figure 1:**
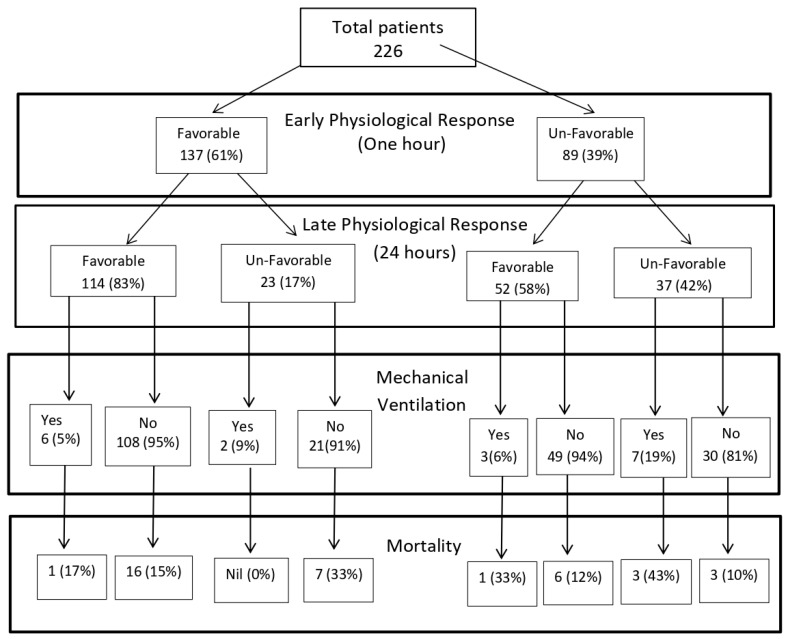
Flow chart showing outcome of the total population in terms of early and late physiological response, need for mechanical ventilation, and mortality.

Figure 1 shows the outcomes of the total population in terms of early and late physiological responses, the need for mechanical ventilation, and mortality. A total of 137/226 patients (61%) showed an early favorable physiological response. After 24 h, 114/137 (83%) had a sustained favorable response, whereas 23/137 (17%) showed an unfavorable response. Out of the 89 (39%) patients showing unfavorable response at one hour, 52 (58%) showed a favorable response at 24 h, while 37/89 (43%) persistently showed unfavorable response. A total of 18 patients were put on mechanical ventilation. The mortality data show that 5 patients on mechanical ventilation died, whereas mortality was seen in 32 patients not put on mechanical ventilation.

Table 2 shows the correlations between initial ABG parameters and IPAP. Initial pH showed a significant negative correlation with initial PCO_2_, while initial PCO_2_ showed a significant positive correlation with bicarbonate levels. Initial PO_2_ showed significant positive correlations with initial PCO_2_ and HCO_3_, and a significant negative correlation with 24 h pH. No significant correlation was seen between IPAP and initial pH; however, there was a significant negative correlation between IPAP and 24 h pH.

Table 3 shows the comparison of factors associated with early and late physiological responses in all disease groups. Initial PCO_2_ showed a significant association with early physiological response. A lower initial PCO_2_ increased the chances of a favorable response at one hour (*p* = 0.001). However, higher initial PO_2_ was associated with fewer chances of late physiological response (*p* = 0.02). Patients with early favorable physiological response had higher initial bicarbonate levels than those showing unfavorable response (*p* = 0.002), but showed no effect on late response. One-hour pH was significantly low at one hour in patients showing unfavorable response (*p* < 0.001), and the same trend was seen in one-hour PCO_2_ (*p* = 0.03). Late favorable physiological response was also seen in patients who required lesser IPAP (*p* < 0.001). Favorable early response and the need for mechanical ventilation were significantly related to late favorable physiological response (*p* < 0.001, *p* = 0.02, respectively). Patients’ clinical and laboratory parameters, pulmonary hypertension, cor pulmonale, and other co-morbid conditions did not affect early and late physiological responses.

Overall mortality was seen in 37 (16%) patients. Table 4 shows that older age, higher initial PO_2_, one-hour and 24 h pH, and higher one-hour PCO_2_ were related to higher mortality. Mortality was also higher in the infective group and patients requiring higher IPAP.

**Figure 2:**
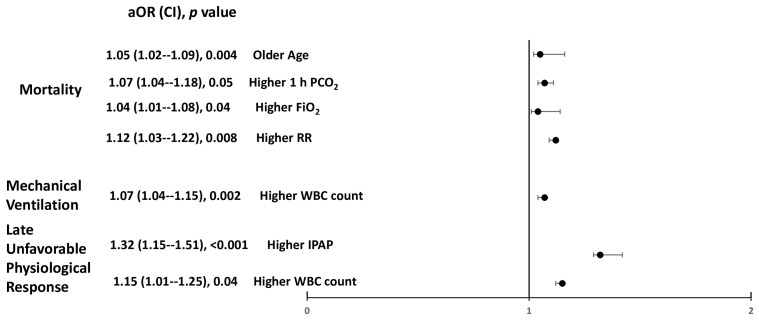
Factors associated with the late unfavorable physiological response, need for mechanical ventilation and mortality of non-invasive ventilation based on binary regression analysis among all patients with hypercapnic respiratory failure with acidosis admitted in the respiratory intensive care unit. PO_2_ = partial pressure of oxygen, PCO_2_ = partial pressure of carbon dioxide, FiO_2_ = fraction of inspired oxygen, RR = respiratory rate, IPAP = inspiratory positive airway pressure, WBC= white blood cell count.

Figure 2 shows the factors associated with late unfavorable physiological response, need for mechanical ventilation, and mortality based on binary regression analysis. Late unfavorable physiological response was associated with high IPAP and white blood cell count. However, mortality was associated with older age, higher PCO_2_ at one-hour, high FiO_2_ requirement, and higher respiratory rate. High white blood cell count predicted the need for mechanical ventilation among these patients.

Supplementary Tables S1–S3 show all factors included in the binary logistic regression model for the late favorable physiological response, mortality, and the need for mechanical ventilation.

## 4. Discussion

Identifying factors associated with early and late unfavorable physiological responses is essential in hospital settings and helps in timely decisions and prompt shifting of patients to mechanical ventilation when required. In our study, severe acidosis at initiation increased the risk of unfavorable response at 24 h; however, no increased risk of unfavorable response was seen at one hour. This finding was probably due to the delayed response to NIV therapy by certain patients. This is consistent with other studies [23,24,31,32,33]; however, as compared to these studies, our studied population included more diverse patients, including obstructive, restrictive, and infective disorders, and was based in the ICU setting rather than the emergency department. Official ERS/ATS guidelines 2017 show that there is no lower limit of pH appropriate for NIV [34]. In our study, if the pH levels after one hour of NIV were higher, chances of sustained response at 24 h were more likely, making one-hour pH a strong predictor of late favorable physiological response, which is also seen in previous studies [35].

Higher initial PCO_2_ levels predict early unfavorable response; however, it was not associated with late physiological response. These findings imply that patients with high initial PCO_2_ levels who cannot clear out CO_2_ within an hour of NIV can clear it later if we continue them on NIV. Previous studies show variable data on the association of levels of PCO_2_ and NIV outcomes. One study showed that high PCO_2_ did not predict NIV failure [31], while another study showed PCO_2_ > 77 mmHg at 1–2 h as a critical predictor of failure [33]. In our study, initial pCO_2_ levels positively correlated with the bicarbonate levels, implying compensation for respiratory acidosis. Thus, high PCO_2_ with metabolic compensation is not a predictor of NIV outcome at one hour. Patients with low bicarbonate levels or concomitant metabolic acidosis did not show a favorable response at one hour. However, the same correlation was not observed at 24 h.

Another significant predictor of unfavorable physiological response was IPAP given to wash out PCO_2_. Relatively higher IPAP was observed in patients showing an unfavorable response at 24 h. However, a literature review shows lower mortality if a higher IPAP is used initially. Our data did show a correlation of IPAP with mortality, but this association was not seen after binary regression analysis [35]. BTS guidelines 2016 also support the use of higher IPAP [25]. The application of higher initial IPAP strongly depends on patients’ tolerance and requires vigilant clinical monitoring. It is evident that patients with better tolerance to NIV have a higher likelihood of favorable outcomes, while those with more severe disease may require higher pressures. Furthermore, patients of older age had a greater likelihood of unfavorable response, which can be easily correlated with poor physiological reserves and severe disease [36].

Patients with higher initial PO_2_ levels had a lower chance of late favorable physiological response. This trend is most likely due to the reduction in hypoxic ventilatory drive, worsening of ventilation-perfusion (V/Q) mismatch, and the Haldane effect, causing worsening of respiratory failure, accumulation of PCO_2_, and worsening of acidosis [37,38,39]. Therefore, controlled oxygen therapy has been a well-known concept for quite a long time. However, chances of success decreased to half at one hour if PO_2_/FiO_2_ is less than 200, representing a higher degree of hypoxia and disease severity. This effect is more extensively studied in NIV use in acute respiratory distress syndrome [40], and used as a marker of severity in ARF [41]. However, this relation was not seen in the binary regression analysis

High WBC counts were associated with poor physiological response and a greater need for mechanical ventilation in our study. In pneumonia, high WBC counts are a well-known predictor of poor outcome in a previous study [42], implying that the comorbidity with infection in obstructive and restrictive disorders can contribute to unfavorable outcomes. However, our mechanically ventilated group includes a small number of patients, so it should be interpreted cautiously. Respiratory rate is also well known marker of disease severity in respiratory disease and part of composite scores [43]. In our study higher respiratory rate was a predictor of poor physiological response at 24 h. This is likely due to the severity of exacerbation and early muscle fatigue in these patients.

Our study showed the association of mortality with higher FiO_2_ requirements. Higher FiO_2_ can affect mortality in two different ways. Firstly, high oxygen requirement represents a higher degree of hypoxia and disease severity, which is likely to affect mortality, as shown in other studies as well. Secondly, hyperoxia can also have a detrimental effect on respiratory physiology. This effect is due to cellular damage by free radicals, leading to worsening of gas exchange, decreased ciliary efficacy, hyperoxic bronchitis, and atelectasis [44,45,46].

There are certain limitations considering the retrospective nature of the study. We lacked data on certain patients’ parameters, which can have an association with NIV failure, such as exhaled tidal volume, leakage, minute ventilation, respiratory symptoms, severity of illness, consciousness, circulatory status, NIV tolerance and drainage of respiratory secretions. Moreover, data on do not intubate decision was also not available. Disease categorization was based on the available data. This can lead to some misclassification between the three types of categories. The grouping of asthma and bronchiectasis under the obstructive category was based on shared obstructive physiology. Considering very few patients in the infective group, more data is required to analyze this subset. Moreover, it is a single-center study with a relatively small sample size and a heterogeneous population, affecting the generalizability of the findings. A prospective study with multicenter cohorts, with the inclusion of more parameters, will be the way forward to expanding research in this area.

## 5. Conclusions

Among patients with diverse respiratory disorders admitted to the respiratory ICU for acute hypercapnic respiratory failure with respiratory acidosis, a favorable physiological response was negatively associated with higher IPAP and WBC count. Higher mortality was seen in elderly patients and in those presenting with a higher one-hour PCO_2_, FiO_2,_ and increased respiratory rate. Elevated WBC count was associated with an increased need for mechanical ventilation in the total study population. Identifying factors contributing to NIV outcomes can help us predict the need for mechanical ventilation in a timely manner. Locally generated data can inform patient selection for initial NIV therapy in resource-limited settings.

## Figures and Tables

**Table 1:** Demographic and clinical characteristics of patients receiving non-invasive ventilation support for acute hypercapnic respiratory failure with acidosis, according to the disease category.

Parameters	Total Patients N = 226	Obstructive Disorders N = 160	Restrictive Disorders N = 56	Infective Disorders N = 10	*p*-Value *
Age (years), median (range)	56 (16–95)	60 (16–95)	45 (25–75)	60 (37–75)	0.001
Gender, n (%)					
Male	139 (62)	112 (70)	22 (39)	5 (50)	<0.001
Female	87 (38)	48 (30)	34 (61)	5 (50)	
BMI (kg/m^2^), median (range)	24 (16–39)	24 (16–43)	23 (18–39)	25 (18–32)	0.660
Smoking, n (%)					
Yes	118 (52)	102 (64)	14 (25)	2 (20)	<0.001
No	108 (48)	58 (36)	42 (75)	8 (80)	
Pulse/min, median (range)	98 (60–190)	97 (60–190)	97 (72–150)	104 (80–142)	0.260
Respiratory rate/min, median (range)	24 (17–47)	24 (17–39)	26 (20–47)	28 (20–40)	0.004
Systolic Blood pressure (mmHg), median (range)	130 (70–160)	120 (70–160)	130 (80–150)	115 (80–140)	0.911
Hemoglobin (g/dL), median (range)	12 (5–20)	12 (5–20)	12 (8–16)	12 (9–14)	0.170
FiO_2_, median (range) ABG Parameters	0.41 (0.25–1.0)	0.41 (0.25–1.0)	0.45 (0.29–0.90)	0.47 (0.41–0.67)	0.010
Initial pH, median (range)	7.26 (6.9–7.38)	7.26 (6.9–7.38)	7.27 (7.08–7.37)	7.22 (7.10–7.32)	0.300
Initial PO_2_ (mmHg), median (range)	66 (31–199)	67 (37–218)	61 (31–181)	54 (36–120)	0.400
Initial PCO_2_ (mmHg), median (range)	82 (47–157)	84 (47–193)	82 (49–157)	60 (49–109)	0.050
Initial HCO_3_ (mEq/L), median (range)	33 (15–82)	34 (18–115)	35 (20–78)	22 (15–40)	0.020
Radiological findings, n (%)					
Hyperinflation	82 (36)	81 (51)	1 (2)	0 (0)	
Consolidation	51 (23)	32 (20)	10 (18)	9 (90)	
Fibrosis	47 (21)	17 (11)	30 (54)	0 (0)	
Mortality, n (%)	37 (16)	25 (16)	6 (11)	5 (50)	0.008
Echocardiography Findings, n (%)	N =109	N = 73	N = 34	N = 2	
Only Pulmonary Hypertension (PHTN)	54 (50)	37 (51)	16 (47)	1 (50)	
PHTN with cor pulmonale	25 (23)	17 (23)	8 (24)	0 (0)	

n = number, N = total number, % = percentage, BMI = Body mass index, FiO_2_ = fraction of inspired air, PO_2_ = partial pressure of oxygen, PCO_2_ = partial pressure of carbon dioxide, HCO_3_ = bicarbonate. An Independent sample Kruskal–Wallis test was used to compare different disease groups’ continuous variables. A chi-square test was used to compare the categorical variables in these groups. * A *p*-value ≤ 0.05 was considered significant. Echocardiography was performed on a limited number of patients (N = 109).

**Table 2:** Pearson correlation of initial pH with initial PCO_2_, initial PO_2_, initial HCO_3_, 24 h pH, and IPAP among all patients receiving non-invasive ventilation support for acute hypercapnic respiratory failure with acidosis in the respiratory intensive care unit.

	Initial pH	Initial PCO_2_	Initial PO_2_	Initial HCO_3_	24 h pH	IPAP
**Initial** **pH**	1					
**Initial PCO_2_**	−0.362 **	1				
**initial PO_2_**	−0.051	0.149 *	1			
**Initial HCO_3_**	0.033	0.819 **	0.197 **	1		
**24 h pH**	0.157 *	−0.022	−0.163 *	0.037	1	
**IPAP**	−0.104	0.081	0.065	−0.017	−0.251 **	1

PCO_2_ = partial pressure of carbon dioxide, PO_2_ = partial pressure of oxygen, HCO_3_ = bicarbonate, IPAP = Inspiratory positive airway pressure. *. Correlation is significant at the 0.05 level (2-tailed). **. Correlation is significant at the 0.01 level (2-tailed).

**Table 3:** Factors associated with the physiological response of non-invasive ventilation support among all patients with hypercapnic respiratory failure with acidosis at one and 24 h in the respiratory intensive care unit.

Parameters	Early Physiological Response		*p* Value	Late Physiological Response		*p*-Value
	Favorable (N = 137)	Unfavorable (N = 145)		Favorable (N = 166)	Unfavorable (N= 60)	
**Age (years)**, median (range)	57 (16–90)	55 (16–95)	0.58	55 (16–90)	57 (25–95)	0.50
**Initial pH**, median (range)	7.24 (6.9–7.38)	7.27 (7.10–7.37)	0.18	7.26 (6.9–7.38)	7.24 (7.10–7.37)	**0.02**
**Initial PCO_2_ (mm/Hg)**, median (range)	75 (49–193)	88 (47–193)	**0.001**	82 (47–173)	83 (48–193)	0.94
**Initial PO_2_**, **(mm/Hg)**, median (range)	64 (31–218)	68 (36–206)	0.23	64 (31–197)	72 (41–218)	**0.02**
**Initial HCO_3_ (mEq/L)**, median (range)	36 (15–115)	31 (18–115)	**0.002**	34 (18–94)	31 (15–115)	0.22
**1 h pH**, median (range)				7.33 (7.07–7.50)	7.25 (7.12–7.53)	**<0.001**
**1 h PCO_2_ (mm/Hg)**, median (range)				68 (12–132)	79 (31–172)	**0.03**
**IPAP (cm H_2_O)**,median (range)	24 (12–30)	24 (15–30)	0.12	22 (12–30)	24 (14–30)	**<0.001**
**EPAP**, **(cm H_2_O)**, median (range)	8 (5–10)	8 (5–12)	0.89	8 (5–12)	8 (5–10)	0.09
**FiO_2_ (%)**, median (range)	41 (25–100)	45 (29–81)	0.12	41 (25–100)	45 (29–90)	0.09
**PO_2_/FiO_2_ (mmHg)**, median (range)	149 (50–565)	149 (59–417)	0.92	146 (50–565)	162 (62–417)	0.31
**Respiratory Rate (/min)**, median (range)	24 (17–40)	24 (20–47)	0.98	24 (17–47)	26 (20–35)	0.97
**Systolic Blood Pressure (mmHg),** median (range)	120 (80–160)	125 (70–150)	0.43	121 (70–160)	125 (90–150)	0.58
**Hemoglobin (gm/dL**) median (range)	12.3 (4.9–19.7)	12.5 (6.8–19.7)	0.87	12.1 (4.9–19.7)	12.6 (9.5–18.8)	0.23
**White blood cell count (** **cells/µL)** **, median (range)**	10.9 (1.7–52)	14 (4.5–47.7)	0.09	11.8 (1.7–47.7)	13.6 (4.5–52)	0.18
**Duration of NIV (days)**, median (range)	5 (1–23)	6 (1–25)	0.39	5 (1–23)	6 (1–25)	0.19
**PHTN**, n/N (%)						
Yes	45/66 (68)	33/42 (78)	0.24	55/81 (68)	23/27 (85)	0.09
No	21/66 (32)	09/42 (22)		26/81 (32)	4/27 (15)	
**Cor pulmonale**, n/N (%)						
Yes	32/45 (71)	21/33 (64)	0.48	32/55 (58)	17/23 (74)	0.35
No	13/45 (29)	12/33 (36)		23/55 (42)	6/23 (26)	
**≥2 comorbs**, n (%)						
Yes	24 (18)	19 (21)	0.62	30 (18)	13 (22)	0.71
No	113 (82)	70 (79)		136 (82)	47 (78)	
Early Favorable Physiological Response						
Yes	-	-	-	116 (42)	23 (20)	**<0.001**
No	-	-		52 (58)	37 (80)	
Mechanical Ventilation, n (%)						
Yes	8 (6)	10 (9)	0.14	9	9	**0.02**
No	129 (94)	79 (91)		157	51	
**Mortality**, n (%)						
No	114 (83)	78 (88)	0.31	144 (85)	48 (80)	0.21
Yes	23 (17)	11 (12)		22 (15)	12 (20)	

NIV = Noninvasive ventilation, n = number, N = total number, min = minute, FiO_2_ = fraction of inspired air, PO_2_ = partial pressure of oxygen, PCO_2_ = partial pressure of carbon dioxide, HCO_3_ = serum bicarbonate, IPAP = Inspiratory positive airway pressure, EPAP = Expiratory positive airway pressure, PHTN = pulmonary hypertension, comorbs = Comorbidities. The Chi-square test and Mann–Whitney U were used for group comparisons. A *p*-value ≤ 0.05 was considered significant.

**Table 4:** Factors associated with mortality among all patients with acute hypercapnic respiratory failure with acidosis receiving non-invasive ventilation support in the respiratory intensive care unit.

Parameter	Survived (N = 189)	Died (N = 37)	*p*-Value
**Age (years)**, n (%)	55 (16–95)	60 (27–86)	0.004
**Initial pH**, median (range)	7.26 (6.9–7.37)	7.25 (7.05–7.38)	0.21
**Initial PCO_2_ (mmHg)**, median (range)	83 (47–173)	76 (49–193)	0.94
**Initial PO_2_ (mmHg)**, median (range)	64 (31–199)	71 (46–218)	**0.02**
**Initial HCO_3_ (mEq/L)**, median (range)	34 (18–94)	30 (15–115)	0.22
**1 h pH**, median (range)	7.32 (7.07–7.50)	7.32 (7.14–7.53)	**<0.001**
**1 h PCO_2_ (mmHg)**, median (range)	64 (12–167)	73 (38–172)	**0.03**
**24 h pH**, median (range)	7.39 (7.20–7.60)	7.39 (6.77–7.60)	**0.001**
**24 h PCO_2_ (mmHg)**, median (range)	62 (28–112)	59 (34–117)	0.32
**Early Physiological response**, n (%)			
Favorable	65 (34)	16 (43)	0.30
Unfavorable	124 (66)	21 (57)	
**Late Physiological response**, n (%)			
Favorable	141 (75)	25 (68)	0.21
Unfavorable	48 (25)	12 (32)	
**IPAP (cmH_2_O)**, median (range)	24 (12–30)	24 (14–30)	**<0.001**
**EPAP (cm H_2_O)**, median (range)	8 (5–12)	8 (6–10)	
**FiO_2_ (%)**, median (range)	41 (25–100)	46 (35–90)	0.09
**PO_2_/FiO_2_ (mmHg),** median (range)	146 (50–565)	158 (67–488)	0.33
**Respiratory Rate (/min)**, median (range)	24 (17–40)	28 (20–47)	0.97
**WBC Cells (/µL),** median (range)	11.8 (3.7–52)	14 (1.7–38)	0.18
**PHTN**, n/N (%)			
Yes	74/102 (73)	4/7 (57)	0.36
No	27/102 (27)	3/7 (43)	
**Cor pulmonale**, n (%)			
Yes	22/75 (29)	3/4 (75)	0.06
No	53/75 (71)	1/4 (25)	
**Disease Category**			
Obstructive Disorder			
Yes	134 (71)	26 (70)	0.34
No	55 (29)	11 (30)	
Restrictive Disorder			
Yes	50 (26)	6 (16)	0.29
No	139 (74)	31 (84)	
Infective Disorder			
Yes	5 (3)	5 (14)	**0.03**
No	184 (97)	32 (86)	

n = number, N = total number, min = minute, WBC = white blood cells, FiO_2_ = fraction of inspired air, PO_2_ = partial pressure of oxygen, PCO_2_ = partial pressure of carbon dioxide, HCO_3_ = bicarbonate, IPAP = inspiratory positive airway pressure, EPAP = Expiratory positive airway pressure, cmH_2_O = centimeter of water, PHTN = pulmonary hypertension. The Mann–Whitney U and chi-square test were used for group comparisons. A *p*-value ≤ 0.05 was considered significant.

## Data Availability

The Supplementary Material provides supporting data. The authors will provide any additional data upon request.

## Supplementary Materials

The following supporting information can be downloaded at: https://www.mdpi.com/article/10.3390/jcm15051701/s1, Supplementary Table S1: Factors associated with mortality among all patients with hypercapnic respiratory failure with acidosis receiving non-invasive ventilation support in the respiratory intensive care unit., Supplementary Table S2: Factors associated with need of mechanical ventilation in patients receiving non-invasive ventilation support for hypercapnic respiratory failure in the respiratory intensive care unit. and Supplementary Table S3: Factors associated with late physiological response (pH ≥7.35 at 24 h) in patients receiving non-invasive ventilation support for hypercapnic respiratory failure in the respiratory intensive care unit.

## Abbreviations

Acute respiratory failure: ARF; Non-invasive ventilation: NIV; chronic obstructive lung disease: COPD; Intensive care unit: ICU; Arterial blood gases: ABGs; Inspiratory positive airway pressure: IPAP.
