# Supplementary material for: Factors Associated with Outcomes of Non-Invasive Ventilation in Acute Hypercapnic Respiratory Failure with Acidosis: A Study from a Tertiary Care Hospital in Pakistan
Source: J Clin Med. 2026 Feb 24;15(5):1701. doi: 10.3390/jcm15051701 (PMC12985644; doi:10.3390/jcm15051701)
Supplement: Supplementary file 1 [file jcm-15-01701-s001.zip › jcm-4024457-supplementary.pdf]

Supplementary Table 1: Factors associated with mortality among all patients with hypercapnic respiratory failure with acidosis receiving non-invasive ventilation support in the respiratory intensive care unit.

**Parameters**	**Survived (N=189)**	**Died (N=37)**	**aOR (95% CI)**	***p* value**
Age (years), median (range)	55 (16-95)	60 (27-86)	1.05 (1.02-1.09)	0.004
Initial pH, median (range)	7.26 (6.9-7.37)	7.25 (7.05-7.38)	1.68 (0.58-4.80)	0.34
Initial PCO_2 (mmHg), median (range)	83 (47-173)	76 (49-193)	0.99 (0.97-1.01)	0.61
Initial PO_2 (mmHg), median (range)	64 (31-199)	71 (46-218)	0.99 (0.99-1.02)	0.76
Initial HCO_3 (mEq/L), median (range)	34 (18-94)	30 (15-115)	1.06 (0.99-1.13)	0.11
1 h pH, n (%)	7.32 (7.07-7.50)	7.32 (7.14-7.53)	28 (0.19-45.00)	0.37
1 h PCO_2 (mmHg), median (range)	64 (38-172)	73 (12-167)	1.07 (1.04-1.18)	0.05
24 h pH, median (range)	7.39 (7.20-7.60)	7.39 (6.77-7.60)	0.03 (0.01-6.07)	0.21
24 h PCO_2 (mmHg), median (range)	62 (28-112)	59 (34-117)	0.99 (0.95-1.03)	0.58
IPAP (cm H2O), median (range)	24 (12-30)	24 (14-30)	1.05 (0.91-1.23)	0.48
FiO_2 (%), median (range)	41 (25-100)	46 (30-90)	1.04 (1.01-1.08)	0.04
PO_2/FiO_2 (mmHg), median (range)	146 (50-565)	158 (67-488)	1.01 (0.99-1.14)	0.19
Systolic Blood Pressure (mmHg), median (range)	121 (80-160)	125 (70-150)	0.98 (0.96-1.01)	0.23
Respiratory Rate(/min), median (range)	24 (17-40)	28 (20-47)	1.12 (1.03-1.22)	0.008
White Cell Count (cells/μL), median (range)	11.8 (3.7-52)	14 (1.7-38)	1.01 (0.95-1.07)	0.62
Disease category, n (%)				
Obstructive				
Restrictive	135	25	0.80 (0.38-1.68)	0.57
Infective	50	06		
	07	03		

Early Physiological response				
Favorable	64	14	1.52 (0.37-6.2)	0.56
Un- Favorable	125	23		
Late Favorable Physiological response				
Favorable	141	2	1.45 (0.36-5.8)	0.59
Un- Favorable	48	15		

n =number, N= total number, FiO_2= fraction of inspired air, PO_2= partial pressure of oxygen, PCO_2= partial pressure of carbon dioxide, HCO_3= serum bicarbonate, IPAP= Inspiratory positive airway pressure, cm H_2O= centimeter of water. A *p* value ≤ 0.05 is considered significant. Binary Logistic Regression was used for two group comparisons.

Supplementary Table 2: Factors associated with need of mechanical ventilation in patients receiving non-invasive ventilation support for hypercapnic respiratory failure in the respiratory intensive care unit.

**Parameter**	**Mechanical ventilation**		**aOR (95% CI)**	***p* value**
	**Yes (N=18)**	**No (N=208)**		
Age (years), median (range)	52 (40-87)	56 (16-95)	0.98 (0.93-1.04)	0.60
Initial pH, median (range)	7.25 (6.9-7.37)	7.26 (7.05-7.38)	0.81 (0.16-4.69)	0.88
Initial PCO_2 (mmHg), median (range)	89 (61-157)	80 (47-197)	0.99 (0.95-1.04)	0.77
Initial PO_2 (mmHg), median (range)	64 (40-141)	65 (31-218)	1.01 (0.96-1.06)	0.67
Initial HCO_3 (mEq/L), median (range)	41 (18-62)	33 (15-115)	1.03 (0.95-1.12)	0.50
1 h pH, n (%)	7.31 (7.07-7.46)	7.32 (7.10-7.53)	2.70 (0.25-12.27)	0.42
1 h PCO_2 (mmHg), median (range)	83 (44-167)	70 (12-172)	0.96 (0.92-1.01)	0.09
24 h pH, median (range)	7.34 (7.21-7.50)	7.39 (6.77-7.60)	2.70 (0.25-29.28)	0.41
24 h PCO_2 (mmHg), median (range)	70 (31-110)	62 (28-117)	1.01 (0.95-1.06)	0.86
IPAP (cm H2O), median (range)	24 (14-26)	24 (12-30)	1.22 (0.96-1.54)	0.09
FiO_2 (%), median (range)	41 (29-90)	41 (25-100)	0.99 (0.93-1.05)	0.67
PO_2/FiO_2 (mmHg), median (range)	153 (62-144)	149 (50-565)	0.99 (0.98-1.02)	0.91

Systolic Blood Pressure (mmHg), median (range)	120 (90-150)	124 (70-160)	0.98 (0.96-1.04)	0.92
Respiratory Rate (/min), median (range)	26 (22-35)	24 (17-47)	0.96 (0.84-1.11)	0.59
White Cell Count cells/μL, median (range)	16.0 (4.7-52.0)	12 (1.7-47.7)	1.07 (1.04-1.15)	0.002
Disease category, n (%)				
Obstructive	13 (72)	146 (70)		
Restrictive	02 (11)	55 (26)	2.90 (0.66-12.89)	0.16
Infective	03 (17)	07 (4)		
Early physiological response				
Favorable	08	129	0.86 (0.17-4.29)	0.85
Un- Favorable	10	79		
Late physiological response				
Favorable	09	157	0.121 (0.01-1.51)	0.10
Un- Favorable	09	51		

n =number, N= total number, FiO_2= fraction of inspired air, PO2= partial pressure of oxygen, PCO_2= partial pressure of carbon dioxide, HCO_3= serum bicarbonate, IPAP= Inspiratory positive airway pressure, cmH_2O= centimeter of water. A *p* value ≤ 0.05 is considered significant. Binary Logistic Regression was used for two group comparisons.

Supplementary Table 3: Factors associated with late physiological response (pH ≥7.35 at 24 hours) in patients receiving non-invasive ventilation support for hypercapnic respiratory failure in the respiratory intensive care unit.

**Parameters**	**Late Physiological Response**		**aOR (95% CI)**	***p* value**
	**Favorable (N=166)**	**Un-favorable (N=66)**		
Age in years, median (range)	55 (16-90)	57 (25-95)	1.02 (0.99-1.05)	0.21
Initial pH, median (range)	7.26 (6.9-7.38)	7.24 (7.10-7.37)	1.60 (0.67-3.84)	0.29
Initial PCO_2 (mmHg), median (range)	82 (47-173)	83 (48-193)	1.00 (0.97-1.04)	0.82
Initial PO_2 (mmHg), median (range)	64 (31-197)	72 (41-218)	0.99 (0.97-1.02)	0.95
Initial HCO_3 (mEq/L), median (range)	34 (18-94)	31 (15-115)	0.96 (0.93-1.01)	0.13
1 Hour PCO_2 (mmHg), median (range)	68 (12-132)	79 (31-172)	1.02 (0.99-1.05)	0.07

IPAP (cm H_2O), median (range)	22 (12-30)	24 (14-30)	1.32 (1.15-1.51)	< 0.001
FiO_2 (%), median (range)	41 (25-100)	45 (29-90)	1.01 (0.97-1.05)	0.55
PO_2/FiO_2 (mmHg), median (range)	146 (50-565)	162 (62-417)	1.00 (0.99-1.01)	0.40
Systolic Blood Pressure (mmHg), median (range)	121 (70-160)	125 (90-150)	1.00 (0.98-1.02)	0.90
Respiratory Rate (/min), median (range)	24 (17-47)	26 (20-35)	0.95 (0.87-1.03)	0.22
White Cell Count (cells/μL), median (range)	11.8 (1.7-47.7)	13.6 (4.5-52)	1.15 (1.01-1.25)	0.04
Disease category, n (%)				
Obstructive	116	44		
Restrictive	45	11	1.40 (0.73-2.77)	0.31
Infective	05	05		
Early physiological response				
Favorable	52	37	0.44 (0.17-1.15)	0.09
Un- Favorable	114	23		

n =number, N= total number, FiO_2= fraction of inspired air, PO2= partial pressure of oxygen, PCO_2= partial pressure of carbon dioxide, HCO_3= serum bicarbonate, IPAP= Inspiratory positive airway pressure, cmH_2O= centimeter of water. A *p* value ≤0.05 is considered significant. Binary Logistic Regression was used for two group comparisons.
